# Perception of and Vaccine Readiness towards Mpox among Men Who Have Sex with Men Living with HIV in China: A Cross-Sectional Study

**DOI:** 10.3390/vaccines11030528

**Published:** 2023-02-23

**Authors:** Leiwen Fu, Yinghui Sun, Yuwei Li, Bingyi Wang, Luoyao Yang, Tian Tian, Xinsheng Wu, Xin Peng, Qi Liu, Yuanyi Chen, Yi-Fan Lin, Hui Li, Xiaojun Meng, Huachun Zou

**Affiliations:** 1 School of Public Health (Shenzhen), Sun Yat-sen University, Shenzhen 518107, China; 2 Center for Disease Control and Prevention, Shizhong District, Jinan 250004, China; 3 Wuxi Municipal Center for Disease Control and Prevention, Wuxi 214023, China

**Keywords:** Mpox, HIV, men who have sex with men, perception, willingness of vaccination

## Abstract

Background: Men who have sex with men (MSM) living with HIV make up the majority of cases in the current Mpox outbreak. We aimed to investigate the perception of and vaccine readiness towards Mpox among MSM living with HIV in China. Methods: This cross-sectional online study was conducted from 10 August to 9 September 2022. Participants responded to survey questions about their socio-demographic information, HIV status, sexual behaviors, knowledge of Mpox, and attitudes towards Mpox vaccines. Results: A total of 577 MSM living with HIV participated in the study. A total of 37.6% expressed concerns about the Mpox epidemic in China, and 56.8% were willing to get the Mpox vaccine. Men who had > four sexual partners in the previous three months (aOR = 1.9 95% CI: 1.2–2.8 Ref: 0), had close contact with > four individuals in a day (3.1, 1.5–6.5 Ref: 0–3), were worried about the Mpox epidemic in China (1.6, 1.1–2.3 Ref: No), and believed that Mpox vaccines are safe (6.6, 2.7–16.4 Ref: No or not sure) and effective (1.9, 1.1–3.3 Ref: No) for people living with HIV were more likely to be willing to get the Mpox vaccine. MSM living with HIV with a high school education or below (0.5, 0.3–0.9 Ref: Postgraduate diploma), and sometimes (0.5, 0.3–0.8 Ref: Often), seldom, or never (0.5, 0.3–0.9 Ref: Often) followed news about Mpox were unwilling to get the Mpox vaccine. Conclusion: The ongoing Mpox pandemic has not attracted widespread concerns among MSM living with HIV in China. Having more sexual partners and close contacts, worrying about the Mpox epidemic, and believing in the vaccine’s safety and efficacy were predictors of their willingness to get the Mpox vaccine. Efforts should be made to raise awareness of the potential risk of Mpox in this at-risk population. Public health strategies should fully address predictors of vaccination willingness.

## 1. Introduction

Human Mpox is a zoonotic disease caused by the Mpox virus (MPXV), first recorded in the Democratic Republic of Congo in 1970 [1]. MPXV is a double-stranded DNA virus similar to smallpox and belongs to the orthopoxvirus [2,3]. Previously, the occurrence of Mpox outside Africa was mostly related to international travel and animal contact, and no secondary human-to-human transmission was confirmed [3,4]. However, since early May 2022, European and North American countries have reported a large number of Mpox cases [5,6,7]. This unusual Mpox outbreak has covered 103 countries and regions worldwide, 96 of which have historically never reported Mpox cases [8]. On 23 July 2022, the World Health Organization (WHO) issued the highest level of alert and declared that the global Mpox outbreak constitutes a Public Health Emergency of International Concern (PHEIC) [9].

As of 12 December 2022, a total of 82,503 confirmed cases, including 65 deaths, have been reported globally [8]. Although there is no clear evidence that the Mpox virus can be sexually transmitted, many recent cases in the current outbreak have been linked to sexual activities, especially among men who have sex with men (MSM) [7,10,11]. Potential sexual transmission is supported by lesions that initially occur at the site of sexual contact, including genital and anal lesions [12]. Surveillance data from multiple countries suggest that 28–51% of MSM patients with Mpox are people living with HIV (PLHIV) [13]. Although clinical data on Mpox in PLHIV are limited, studies have described that advanced or uncontrolled HIV infection may result in more severe outcomes. A study from Nigeria showed that PLHIV were more likely to have a larger skin rash, secondary bacterial infection, and longer duration of illness [14]. Recent reports from several countries with Mpox outbreaks indicate that a more severe disease course has not been described in PLHIV who have a robust immune system [15,16]. There is currently no specific treatment for Mpox, and vaccination is recommended for those at high risk of infection. There are two vaccines available to prevent orthopoxvirus infection [13]. JYNNEOS, a live virus vaccine, is licensed by the Food and Drug Administration to protect against Mpox in adults and has been evaluated to be safe and effective in PLHIV [17,18]. ACAM2000 is contraindicated in PLHIV because it may cause serious Mpox-related diseases in people with compromised immune systems [19].

Existing studies have found that many PLHIV continue to be sexually active and engage in risky sexual behaviors after their diagnosis [20,21,22]. We believe that MSM living with HIV are a high-risk population in the current Mpox outbreak. When vaccine resources are limited, MSM living with HIV may need to be considered as a priority group. In addition, previous studies have shown that despite specific guidelines, people living with HIV remain skeptical about the safety and effectiveness of vaccination because of their immunosuppressed status [23,24]. It is necessary to assess the perception of and vaccine readiness towards Mpox among this population who are at very high risk for Mpox transmission. Most studies have been based on the general population’s perception of Mpox, and little attention has been paid to MSM living with HIV [25,26], who comprise the majority of cases in the ongoing Mpox pandemic. This study is one of the first attempts to assess the perceptions of and vaccine readiness towards Mpox among MSM living with HIV in China. Correlates of the acceptance of Mpox vaccination were further tested in this population.

## 2. Methods

### 2.1. Study Design and Participants

This study is reported as per the Strengthening the Reporting of Observational Studies in Epidemiology (STROBE) guidelines (Table S1) [27].

This cross-sectional web-based study was conducted between 10 August and 9 September 2022. Participants were recruited by convenience sampling through Li Hui Shi Kong, an online WeChat official account with over 76,000 subscribers living with HIV from various regions in the country, and the estimated proportion of MSM is 75% [28]. When the survey QR code attached to the poster advertisement was scanned, a voluntary informed consent screen would be first opened. The voluntary informed consent also provided information about the purpose of the study, which was to investigate the perceptions of and vaccine readiness towards Mpox among MSM living with HIV. Participants who acknowledged reading the informed consent and providing consent electronically were directed to the formal questions of the questionnaire. The online questionnaire was programmed on Wenjuanxing, an online questionnaire survey platform with 82 million users in China. Inclusion criteria of individuals included the following: (1) ≥18 years, (2) identified as MSM, (3) self-identified as HIV-positive, and (4) had internet access. Study eligibilities were assessed automatically through the Wenjuanxing platform and double-checked during data cleaning.

### 2.2. Measures

A structured questionnaire was composed of six main sections addressing the following: socio-demographic information, HIV status, sexual behaviors, social life, knowledge of Mpox, and attitudes towards vaccines. The survey instruments used in this study were adapted from our previously published studies on COVID-19 and modified according to the questionnaire of other studies on the knowledge of and attitudes towards the current Mpox outbreak [25,29,30,31,32].

Variables in socio-demographics were as follows: age, gender identity, educational attainment, accommodation location, marital status, employment attainment, salary, sexual orientation, and disease history of chronic diseases and STD infections. People aged 18–25 are considered to be adolescents and young adults, while people over 40 are often classified as middle-aged, and may differ in their sexual behaviors, levels of knowledge, and risk awareness. Therefore, age groups were divided into three groups (≤25, 26–39, and >40) in this study. Chronic disease including cardiovascular disease, cancer, chronic respiratory disease, and other chronic diseases. For health conditions and sexual behaviors, participants reported information including their time since HIV diagnosis, latest HIV viral load results, latest CD+ 4 cell count (per uL), ART adherence (eight-item Morisky Medication Adherence Scale), vaccine history of smallpox, anal and oral sex in the past three months, number of anal sex partners in the past three months, and condom use. Inconsistent condom use was defined as reporting any frequency other than “always” during anal sex in the past three months. We also asked individuals whether they had recently experienced close daily contact, close daily conversations, or attended entertainment events. Close contact was defined as contact with body parts or bodily fluids (e.g., saliva, blood, sweat). Close conversations were defined as verbal communication within 1 m without a mask. Participants answered questions on their perceptions of Mpox, including their information sources and frequency of seeking information about Mpox, concerns about the Mpox epidemic, the safety and effectiveness of the Mpox vaccine, and perceived symptoms and transmission characteristics of Mpox. The willingness to adopt Mpox vaccine was measured by a five-point Likert scale (“Would you like to be vaccinated against Mpox?”) ranging from 1 (strongly disagree) to 5 (strongly agree). For this study, vaccine acceptance was re-categorized as willingness (4–5) and unwillingness (1–3). Participants who were unwilling to be vaccinated were asked to state their main reasons for their unwillingness. The seven-item Generalized Anxiety Disorder Scale (GAD-7) was used to assess anxiety status in this population [29]. The standardized Cronbach’s α of the GAD-7 in this study was 0.949. The Kaiser–Meyer–Olkin measure of sampling adequacy of the GAD-7 in this study was 0.945. Before the questionnaire was distributed, 54 MSM living with HIV participated in a pilot study to verify and modify the contents of the questionnaire. However, these participants in a pilot study were not included in the final survey.

### 2.3. Statistical Analysis

Descriptive analysis was performed for numerical and categorical variables. Additionally, median and interquartile range (IQR) or frequencies were calculated. Pearson’s chi-squared test was used to compare variables between MSM living with HIV willing to or refusing to get the Mpox vaccine. Two-sided *p* < 0.05 is considered statistically significant. Multivariable logistic regression was used to explore the associated factors of participants’ willingness to get the Mpox vaccine. Pearson’s chi-squared test was used to assess what variables could potentially explain participants’ support for vaccination against Mpox. Multivariate modeling was carried out using multiple logistic regression with the final choice of the model, including variables with *p* < 0.05 in univariate analysis. We also performed a multicollinearity diagnosis for the final logistic regression model by using the variance inflation factor (VIF). For any predictor variable, the square root of the VIF indicates the degree to which the confidence interval for that variable’s regression parameter is expanded relative to a model with uncorrelated predictors. As a general rule, a VIF > 10 indicates a multicollinearity problem. We used stepwise logistic regression to identify predictors of willingness to get the Mpox vaccine. Results of final logistic regression were reported as odds ratios (OR) and adjusted odds ratios (aOR) with corresponding 95% confidence intervals (95% CI). Analyses were performed using IBM SPSS Version 20.0 (SPSS, Inc. Chicago, IL, USA).

## 3. Results

### 3.1. Background Characteristics

The online survey was completed by 577 eligible MSM living with HIV, covering seven administrative divisions in mainland China (Table 1). Five hundred and twenty-three (90.6%) participants were cisgender men, and 54 (9.4%) participants were transgender women. The median age of the participants was 32 years (inter-quartile range: 28–37). Most of the participants were single (50.3%) and had a Bachelor’s degree (69.5%). A higher proportion of participants (46.1%) were employed, with most reporting a salary between CNY 5000 and 9999 (USD 722–1444). Seventy-two participants (12.5%) had a self-reported history of chronic disease; the most common sexually transmitted disease self-reported was syphilis (10.4%). More than half of the participants (56.8%) were ready to get the vaccine against Mpox at the current stage of the disease. The participants in the willing group were more likely to have a postgraduate diploma (*p* = 0.028).

Regarding their HIV statuses and sexual behaviors, 371 of the participants (64.3%) had been diagnosed with HIV for over three years, and 443 participants (76.8%) reported an undetectable viral load of HIV (Table 2). The majority of the participants (90.4%) had medium or high ART adherence. A total of 392 participants (67.9%) had anal sex in the past three months, 47.7% reported inconsistent condom use, and 18.9% reported more than four sexual partners. About half of the participants reported anal (45.1%) or oral (51.5%) sex more than once per month. Furthermore, a minority of participants (8.8%) reported having close contact with more than four individuals in a day, and 346 participants (60.0%) reported having close conversations with more than four individuals in a day. Participants in the willing group were more likely to have an undetectable HIV viral load (*p* < 0.001), high ART adherence (*p* = 0.040), fewer anal sex partners in the past three months (*p* < 0.001), and more close contacts daily (*p* = 0.018).

### 3.2. Knowledge about Mpox

As shown in Table 3, one in five (20.8%) participants followed news about Mpox frequently, and the main way they did so was through news media, such as the Internet (79.9%). Two hundred and seventeen participants (37.6%) were worried about the Mpox epidemic in China. Most participants agreed that Mpox could infect anyone through close contact (93.6%), and individuals living with HIV make up a large proportion of the current Mpox outbreak (80.1%). Regarding vaccines, 45.9% of the participants reported having received the smallpox vaccine, while 81.3% agreed that Mpox vaccines were effective against Mpox. Participants in the willing group were more likely to follow news about Mpox often (*p* < 0.001), get vaccinated for smallpox (*p* = 0.029), worry about the Mpox epidemic in China (*p* = 0.007), believe Mpox vaccines are safe for PLHIV (*p* < 0.001), and believe Mpox vaccines can be effective against Mpox (*p* < 0.001).

**Figure 1:**
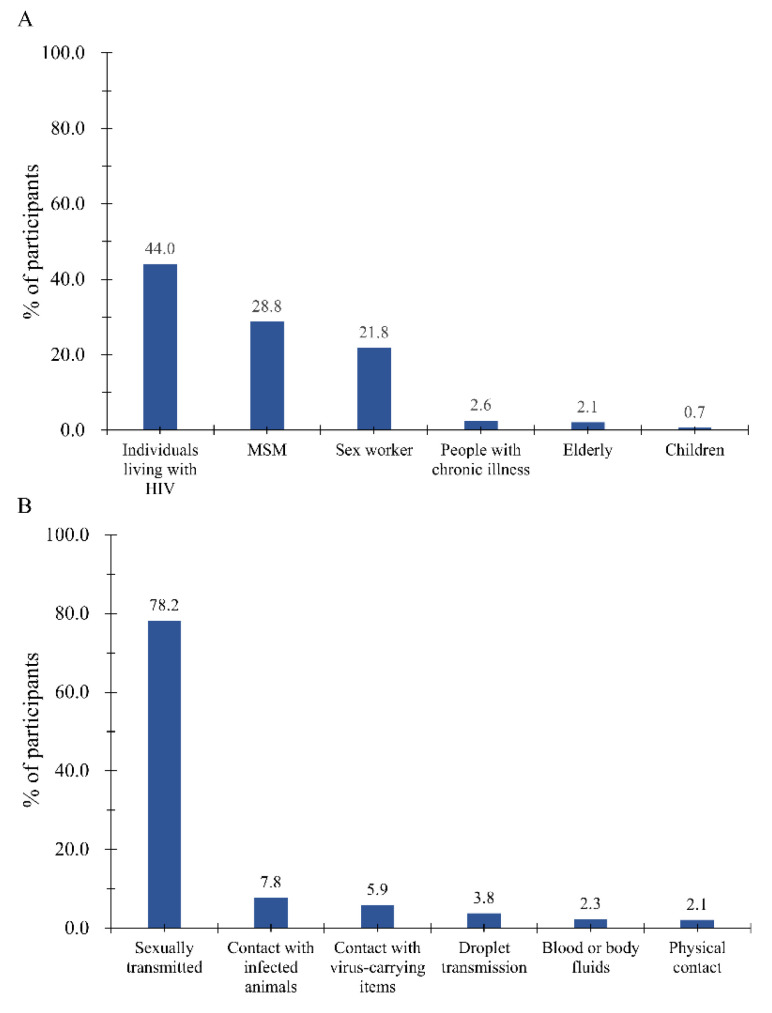
Participates’ perceived groups at high risk (**A**) and dominant transmission route of Mpox (**B**). An elderly person is defined as anyone over the age of 60. A child is defined as anyone under the age of 18.

A total of 44.0% of the participants considered individuals living with HIV to be the highest risk group for Mpox (Figure 1A), followed by MSM (28.8%), sex workers (21.8%), people with chronic illnesses (2.7%), the elderly (2.1%), and children (0.7%). Regarding the dominant transmission route of Mpox in the current outbreak (Figure 1B), most participants considered this to be sexual transmission (78.2%), followed by contact with infected animals (7.8%), contact with virus-carrying items (5.9%), droplet transmission (3.8%), blood or body fluids (2.3%), and physical contact (2.1%).

### 3.3. Reasons for Unwillingness to Get the Mpox Vaccine

**Figure 2:**
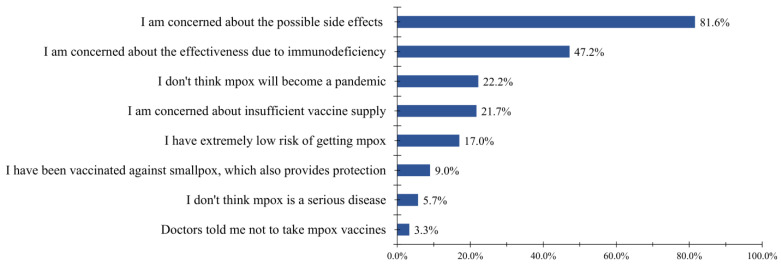
Reasons for unwillingness to get vaccinated against Mpox.

Figure 2 shows the reasons for the participants’ unwillingness to get vaccinated against Mpox. Most participants (81.6%) were concerned about the possible side effects of Mpox vaccines. A total of 47.2% of participants were concerned that the vaccine would not work in an immunocompromised population. Other reasons were as follows: believing that Mpox will not become a pandemic (22.2%), having concerns about insufficient vaccine supply (21.7%), having an extremely low risk of contracting Mpox (17.0%), having been vaccinated against smallpox which also provides protection (9.0%), believing that Mpox is not a serious disease (5.7%), and being told not to take the Mpox vaccine by a doctor (3.3%).

### 3.4. Predictors of Willingness to Get the Mpox Vaccine

Table 4 presents the predictors of the participants’ willingness to get the Mpox vaccine among MSM living with HIV. In total, ten predictors of willingness to get the Mpox vaccine were included in the final model, including one sociodemographic variable (‘Education’), two HIV status variables (‘Latest HIV viral load’ and ‘ART adherence’), one sexual behaviors variable (‘Number of anal sex partners in the past three months’), one social contact variable (‘Self-reported number of close contacts daily’), and five knowledge variables (‘Frequency of following information about the Mpox’, ‘Uptake smallpox vaccination’, ‘Worry about the Mpox epidemic in China’, ‘I believe Mpox vaccines are safe for PLHIV’, and ‘Mpox vaccines can be effective against Mpox’). In the multivariate logistic regression analysis, men who had > four sexual partners in past three months (aOR = 1.9 95% CI: 1.2–2.8 Ref: 0), had close contact with > four individuals in a day (3.1, 1.5–6.5 Ref: 0–3), were worried about the Mpox epidemic in China (1.6, 1.1–2.3 Ref: No), and believed that the Mpox vaccine is safe (6.6, 2.7–16.4 Ref: No or not sure) and effective (1.9, 1.1–3.3 Ref: No) for PLHIV were more willing to get the vaccination. MSM living with HIV who had a high school education or below (0.5, 0.3–0.9 Ref: Postgraduate diploma), and sometimes (0.5, 0.3–0.8 Ref: Often), seldom, or never (0.5, 0.3–0.9 Ref: Often) followed news about Mpox were less willing to get the vaccination. Three variables were no longer statistically significant when entered into the multivariate logistic regression analysis.

## 4. Discussion

Our study found that 37.6% of MSM living with HIV in China expressed concerns about the potential Mpox epidemic in China and 56.8% were willing to get the Mpox vaccine. Willingness to get the Mpox vaccine was associated with educational level, number of sexual partners in the past three months, number of daily close contacts, frequency of seeking news about Mpox, and awareness of Mpox.

Our study showed that 73% of the participants sometimes or frequently followed the news about Mpox, and half of the participants agreed that PLHIV were at a higher risk of Mpox infection. China had not reported any Mpox cases before the current outbreak in 2022, as it used to be endemic to Africa [33]. The higher interest in Mpox among MSM living with HIV may be attributed to the fact that MSM make up the majority of cases in the current multinational Mpox outbreak. Although there is currently no evidence that HIV increases the likelihood of Mpox transmission, PLHIV accounted for approximately 40% of current Mpox cases [7]. However, higher interest in Mpox cannot be interpreted as a concern about the prevalence of Mpox. Our data showed that 37.6% of participants indicated concerns about the current Mpox outbreak spreading to China, which is similar than the findings of the general population in Saudi Arabia (37.4%) [25]. China has implemented strict entry screening measures, and screening for COVID-19 during the quarantine period is accompanied by Mpox testing [34]. Therefore, apart from two imported cases of Mpox in Hong Kong and Chongqing, there are currently no autochthonous cases of Mpox that have been reported in China, so there is no widespread concern [35,36]. Social restrictions have varied from province to province. These restrictions and measures may have prevented Mpox cases from entering the country. However, at the same time, it may have also led to us neglecting the risk of Mpox.

Changes in transmission patterns of the current Mpox outbreak may also partly explain the lower concerns of this population. Previous studies have confirmed the presence of the Mpox virus in semen [37,38,39]. Heskin et al. [12] recently reported a case of Mpox with documented transmission through sex. Furthermore, studies from Italy have documented Mpox co-infection with other sexually transmitted infections (STIs), supporting the hypothesis of sexual transmission [38,40]. The impact of Mpox appears to be more limited than that of COVID-19, which is transmitted through respiratory droplets. Most participants in our survey believed that in this outbreak, the highest risk of Mpox transmission was through sexual encounters, suggesting that this population might have knowledge of novel transmission routes on top of the classic transmission routes identified in this outbreak.

Vaccination is an important tool in preventing the spread of Mpox. A total of 56.8% of MSM living with HIV in our study were willing to get the Mpox vaccine. Similar to our findings, 59.8% of MSM living with HIV in France indicated their acceptance towards Mpox vaccination [41]. Dukers-Muijrers and colleagues found that 81.5% of the MSM surveyed in the Netherlands were willing to receive an Mpox vaccine [42]. However, the willingness rate of Mpox vaccination varied among different populations and regions [43]. In the general population, the willingness rate to get the Mpox vaccine ranged from 29% in Romania to 50.6% in Saudi Arabia [25,44]. Among healthcare workers, the rate of willingness to get the Mpox vaccine varied from 8.8% in the Czech Republic to 90.1% in China [45,46].

Concerns about the possible side effects of Mpox vaccines and concerns about its effectiveness due to immunodeficiency were considered critical to participants’ unwillingness to get vaccinated against Mpox in our study. Lack of faith in the efficacy and safety of vaccines is a major challenge in achieving vaccine coverage among PLHIV, which has been widely reported [47]. Zheng et al. [29] reported that 47.2% of MSM living with HIV had a high level of COVID-19 vaccine hesitancy. A cohort study of HIV patients found that only about 39% of PLHIV got vaccined against influenza [47]. This concern is understandable, given the lack of a global consensus among experts on Mpox vaccination for PLHIV. The interim guidelines for the prevention and treatment of Mpox in person with HIV infection issued by the United States CDC showed that JYNNEOS was well tolerated with similar immunogenicity in individuals with CD4 cell count of 200–750/μL, regardless of HIV infection status [13]. However, immunogenicity remains unknown in PLHIV with CD4 cell counts <100/µL or those who are not virologically suppressed. Due to the current limited availability of the Mpox vaccine, the CDC recommends prioritizing vaccination for those who have been exposed to suspected or confirmed cases of Mpox [48]. For patients living with HIV at risk of severe Mpox infection, clinicians should make reasonable prevention and treatment recommendations based on their clinical status, viral suppression, and CD4 cell count.

Our results demonstrated that participants who had multiple sexual partners and daily close contacts in the past three months were more willing to receive the Mpox vaccine. Ibuka et al. [49] also reported that social contact might affect influenza vaccination status and the probability of vaccination increased with the number of contacts. As Mpox spreads through close contact, participants who have more sexual partners and close contacts were perceived to confront a higher risk of infection. They should take additional precautions to reduce these potential risks or to protect others. This study also showed that participants who believed the Mpox vaccines were safe and effective were more likely to be vaccinated. The frequency of following Mpox news was a predictor of willingness to get the Mpox vaccine, and the Internet was the main source of information. Participants who sometimes and rarely paid attention to information about Mpox were less likely to get vaccinated than those who frequently paid attention. Our colleagues found similar results in COVID-19 vaccine uptake and hesitancy among this population [29]. This may be attributable to limited access to online health resources and caring more for one’s own health [50]. This study also showed that participants with a high school education or below were more likely not to be vaccinated. A recent meta-analysis reported that educational level was a strong predictor of COVID-19 vaccination willingness [51]. Lower education may limit people’s access to health information and may interfere with their judgments.

The current study is one of the few studies that provide insight into Mpox perception and vaccine readiness among MSM living with HIV in China. The pilot study conducted in this study helps to strengthen the logic of the questionnaire and can allow us to more comprehensively understand the suggestions of the population on the questionnaire, with a good quality control effect. Several limitations should be noted. First, the cross-sectional design of this study limits the determination of causality. Second, convenience sampling and non-participation could have introduced some selection bias. The sample in this study might not fully represent all MSM living with HIV in China, and the generalizability of the findings needs to be interpreted with caution. Third, self-reported data may lead to recall and social desirability biases, especially regarding data on sensitive information and on smallpox vaccination. By distributing an anonymous questionnaire, social expectation bias might be reduced. Forth, some measurements of Mpox knowledge and attitudes in this study did not use a scale and the questionnaire did not contain reliability and validity measurements, but referred to those of previous studies. Fifth, the data collection occurred during the COVID-19 pandemic, and this context should be taken into consideration when interpreting the results. Sixth, our study included only participants with Internet access, who may have better educational and economic conditions, which may have led to an overestimation of the willingness to get vaccinated. Finally, participants’ perceptions of the Mpox threat and willingness to get the Mpox vaccine may change as the situation evolves. It is worth noting that there were no Mpox cases reported in mainland China at the time of data collection, until the first imported case was reported in Chongqing, China on 16 September 2022 [36]. Future studies could explore the impact of this factor on Mpox perception in this population.

## 5. Conclusions

The ongoing Mpox pandemic has not attracted widespread concerns among MSM living with HIV in China. Just over half of MSM living with HIV were willing to get the Mpox vaccine. Efforts should be made to raise awareness of the potential risk of Mpox in this at-risk population. Public health strategies should fully address predictors of vaccination willingness.

## Figures and Tables

**Table 1:** Demographic characteristics of MSM living with HIV in China.

Characteristics	Total N = 577	Willing to Receive Mpox Vaccination *n* = 328	Unwilling to Receive Mpox Vaccination *n* = 249	*p* Value
Gender Identity				0.624
Cisgender male	523 (90.6)	299 (91.2)	224 (90.0)	
Transgender women	54 (9.4)	29 (8.8)	25 (10.0)	
Age, year				0.319
≤25	88 (15.3)	44 (13.4)	44 (17.7)	
26–39	400 (69.3)	230 (70.1)	170 (68.2)	
≥40	89 (15.4)	54 (16.5)	35 (14.1)	
Education				0.028
High school or below	103 (17.9)	55 (16.8)	48 (19.3)	
Undergraduate diploma	401 (69.5)	221 (67.4)	180 (72.3)	
Postgraduate diploma	73 (12.7)	52 (15.9)	21 (8.4)	
Chinese geographical division				0.177
North China	80 (13.9)	50 (15.2)	30 (12.0)	
Northeast China	26 (4.5)	17 (5.2)	9 (3.6)	
East China	221 (38.3)	120 (36.6)	101 (40.6)	
Central China	64 (11.1)	46 (14.0)	22 (8.8)	
South China	90 (15.6)	43 (13.1)	47 (18.9)	
Southwest China	68 (11.8)	17 (5.2)	11 (4.4)	
Northwest China	28 (4.9)	35 (10.7)	29 (11.6)	
Marital status				0.412
Single	290 (50.3)	156 (47.6)	134 (53.8)	
Unmarried	219 (38.0)	132 (40.2)	87 (34.9)	
Married	50 (8.7)	28 (8.5)	22 (8.8)	
Other	18 (3.1)	12 (3.7)	6 (2.4)	
Employment status				0.674
Full-time employment	504 (87.3)	290 (88.4)	214 (85.9)	
Student	40 (6.9)	21 (6.4)	19 (7.6)	
Unemployed	33 (5.7)	17 (5.2)	16 (6.4)	
Salary (CNY)				0.286
0–4999	201 (34.8)	107 (32.6)	94 (37.8)	
5000–9999	237 (41.1)	135 (41.2)	102 (41.0)	
≥10,000	139 (24.1)	86 (26.2)	53 (21.3)	
Region				0.270
Urban	534 (92.5)	307 (93.6)	227 (91.2)	
Rural	43 (7.5)	21 (6.4)	22 (8.8)	
Sexual orientation				0.854
Heterosexual or bisexual	79 (13.7)	47 (14.3)	32 (12.9)	
Homosexual	488 (84.6)	275 (83.8)	213 (85.5)	
Other or not sure	10 (1.7)	6 (1.8)	4 (1.6)	
History of chronic diseases				0.598
No	505 (87.5)	285 (86.9)	220 (88.4)	
Yes	72 (12.5)	43 (13.1)	29 (11.6)	
Self-reported STD diagnoses				0.845
Yes	83 (14.4)	48 (14.6)	35 (14.1)	
No	494 (85.6)	280 (85.4)	214 (85.9)	
Level of anxiety severity, GAD-7 score				0.428
No or mild anxiety (<10)	488 (84.6)	274 (83.5)	214 (85.9)	
Moderate to severe anxiety (≥10)	89 (15.4)	54 (16.5)	35 (14.1)	

Abbreviations: CNY: Chinese Yuan; CNY 100 ≈ USD 15; ART: antiretroviral therapy; MSM: men who have sex with men; HIV: human immunodeficiency virus; STD: Sexually transmitted diseases. Chinese Geographical Division: North China (Beijing, Tianjin, Hebei, Shanxi, Inner Mongolia); Northeast China (Heilongjiang, Jilin, Liaoning); East China (Shanghai, Jiangsu, Zhejiang, Anhui, Jiangxi, Shandong, Fujian, Taiwan); Central China (Henan, Hubei, Hunan); South China (Guangdong, Guangxi, Hainan, Hong Kong, Macao); Southwest China (Chongqing, Sichuan, Guizhou, Yunnan, Tibet); Northwest China (Shaanxi, Gansu, Qinghai, Ningxia, Xinjiang). Chronic disease including cardiovascular disease, cancer, chronic respiratory disease, and other chronic diseases.

**Table 2:** HIV status, sexual behaviors, and social contact variables related to willingness to get vaccinated among MSM living with HIV in China.

Variables	Total N = 577	Willing to Receive Mpox Vaccination *n* = 328	Unwilling to Receive Mpox Vaccination *n* = 249	*p* Value
Time since HIV diagnosis (months)				0.969
≤12	78 (13.5)	44 (13.4)	34 (13.7)	
13–35	128 (22.2)	74 (22.6)	54 (21.7)	
≥36	371 (64.3)	210 (64.0)	161 (64.7)	
Latest HIV viral load				<0.001
Detectable	82 (14.2)	41 (12.5)	41 (16.5)	
Undetectable	443 (76.8)	269 (82.0)	174 (69.9)	
Not sure	52 (9.0)	18 (5.5)	34 (13.7)	
Latest CD4 cell count (per uL)				0.268
<200	16 (2.8)	6 (1.8)	10 (4.0)	
200 to 349	62 (10.7)	35 (10.7)	27 (10.8)	
350 to 499	164 (28.4)	97 (29.6)	67 (26.9)	
≥500	307 (53.2)	178 (54.3)	129 (51.8)	
Not sure	28 (4.9)	12 (3.7)	16 (6.4)	
ART adherence, MMAS-8 score				0.040
Low (scores < 6)	55 (9.6)	24 (7.4)	31 (12.6)	
Medium (6 ≤ scores < 8)	187 (32.7)	101 (31.1)	86 (34.8)	
High (scores = 8)	330 (57.7)	200 (61.5)	130 (52.6)	
Anal sex in the past three months				<0.001
No	185 (32.1)	87 (26.5)	98 (39.4)	
Yes	392 (67.9)	241 (73.5)	151 (60.6)	
Frequency of anal sex in the past three months				0.568
≥4 times per month	72 (12.5)	42 (12.8)	30 (12.0)	
1–3 times per month	188 (32.6)	112 (34.1)	76 (30.5)	
<1 time per month	317 (54.9)	174 (53.0)	143 (57.4)	
Frequency of oral sex in the past three months				0.112
≥4 times per month	84 (14.6)	46 (14.0)	38 (15.3)	
1–3 times per month	213 (36.9)	133 (40.5)	80 (32.1)	
<1 time per month	280 (48.5)	149 (45.4)	131 (52.6)	
Number of anal sex partners in the past three months				<0.001
≥4	74 (12.8)	37 (11.3)	37 (14.9)	
1–3	318 (55.1)	204 (62.2)	114 (45.8)	
0	185 (32.1)	87 (26.5)	98 (39.4)	
Inconsistent condom use during anal sex				0.683
Yes	187 (47.7)	113 (46.9)	74 (49.0)	
No	205 (52.3)	128 (53.1)	77 (51.0)	
Experience community lockdown due to COVID-19				0.413
Yes	175 (30.3)	95 (29.0)	80 (32.1)	
No	402 (69.7)	233 (71.0)	169 (67.9)	
Self-reported number of close contacts daily				0.018
0–3	526 (91.2)	291 (88.7)	235 (94.4)	
≥4	51 (8.8)	37 (11.3)	14 (5.6)	
Self-reported number of close conversations daily				0.401
0–3	231 (40.0)	138 (42.1)	93 (37.3)	
4–7	137 (23.7)	72 (22.0)	65 (26.1)	
≥8	209 (36.2)	118 (36.0)	91 (36.5)	
Frequency of visiting entertainment venues in the past three months				0.181
≥4 times per month	24 (4.2)	13 (4.0)	11 (4.4)	
1–3 times per month	39 (6.8)	27 (8.2)	12 (4.8)	
<1 time per month	103 (17.9)	51 (15.5)	52 (20.9)	
Never	441 (71.2)	237 (72.3)	174 (69.9)	

Abbreviations: ART: antiretroviral therapy; MSM: men who have sex with men; HIV: human immunodeficiency virus. ART adherence information is missing for five individuals.

**Table 3:** Knowledge about Mpox variables related to willingness to get vaccinated among MSM living with HIV in China.

Variables	Total N = 577	Willing to Receive Mpox Vaccination *n* = 328	Unwilling to Receive Mpox Vaccination *n* = 249	*p* Value
Frequency of following information about the Mpox *				<0.001
Often	120 (20.8)	89 (27.1)	31 (12.4)	
Sometimes	303 (52.5)	163 (49.7)	140 (56.2)	
Seldom or never	154 (26.7)	76 (23.2)	78 (31.3)	
Source of Mpox information				
TV programs or Newspaper	166 (28.8)	95 (29.0)	71 (28.5)	0.906
Internet	461 (79.9)	261 (79.6)	200 (80.3)	0.824
Communication with friends and family	25 (4.3)	15 (4.6)	10 (4.0)	0.745
Uptake smallpox vaccination				0.029
Yes	265 (45.9)	160 (48.8)	105 (42.2)	
No	213 (36.9)	106 (32.3)	107 (43.0)	
Not sure	99 (17.2)	62 (18.9)	37 (14.9)	
Worry about the Mpox epidemic in China				0.007
Yes	217 (37.6)	139 (42.4)	78 (31.3)	
No	360 (62.4)	189 (57.6)	171 (68.7)	
I believe Mpox vaccines are safe for PLHIV				<0.001
Yes	519 (89.9)	321 (97.9)	198 (79.5)	
No	21 (3.6)	7 (2.1)	14 (5.6)	
Not sure	37 (6.4)	0 (0.0)	37 (14.9)	
Mpox can spread to anyone through close contact				0.084
Yes	540 (93.6)	312 (95.1)	228 (91.6)	
No	37 (6.4)	16 (4.9)	21 (8.4)	
Most people with Mpox recover fully within 2 to 4 weeks without the need for medical treatment				0.646
Yes	333 (57.7)	192 (58.5)	141 (56.6)	
No	244 (42.3)	136 (41.5)	108 (43.4)	
PLHIV make up a large proportion of current Mpox outbreak				0.358
Yes	462 (80.1)	267 (81.4)	195 (78.3)	
No	115 (19.9)	61 (18.6)	54 (21.7)	
ART may reduce the risk of severe illness from Mpox in PLHIV				0.565
Yes	286 (49.6)	166 (50.6)	120 (48.2)	
No	291 (50.4)	162 (49.4)	129 (51.8)	
Mpox vaccines can be effective against Mpox				<0.001
Yes	469 (81.3)	293 (89.3)	176 (70.7)	
No	108 (18.7)	35 (10.7)	73 (29.3)	

Abbreviations: ART: antiretroviral therapy; MSM: men who have sex with men; HIV: human immunodeficiency virus; PLHIV: people living with HIV. * Participants could list all sources of information they used.

**Table 4:** Correlates of Mpox vaccination willingness among MSM living with HIV in China.

Variables	Unadjusted OR (95% CI)	*p* Value	aOR (95% CI)	*p* Value
Education				
High school or below	0.5 (0.3–0.9)	0.011	0.5 (0.3–0.9)	0.041
Undergraduate diploma	0.5 (0.2–0.9)	0.018	0.7 (0.3–1.5)	0.356
Postgraduate diploma	Ref		Ref	
Latest HIV viral load				
Undetectable	Ref		Ref	
Detectable or not sure	0.6 (0.3–0.8)	0.001	0.6 (0.4–1.0)	0.063
ART adherence, MMAS-8 score				
Low (scores < 6)	Ref		Ref	
Medium (6 ≤ scores < 8)	1.5 (0.8–2.8)	0.177	1.1 (0.6–2.3)	0.730
High (scores = 8)	2.0 (1.1–3.5)	0.020	1.7 (0.9–3.2)	0.128
Uptake Smallpox vaccination				
No	Ref		Ref	
Yes	1.5 (1.1–2.2)	0.021	1.3 (0.8–2.0)	0.246
Not sure	1.7 (1.0–2.8)	0.035	1.5 (0.9–2.7)	0.127
Number of anal sex partners in the past three months				
≥4	2.0 (1.4–2.9)	<0.001	1.9 (1.2–2.8)	0.003
1–3	1.1 (0.7–1.9)	0.665	0.8 (0.4–1.5)	0.490
0	Ref		Ref	
Self-reported number of close contacts daily				
0–3	Ref		Ref	
≥4	2.1 (1.1–4.0)	0.020	3.1 (1.5–6.5)	0.003
Frequency of following information about the Mpox				
Often	Ref		Ref	
Sometimes	0.4 (0.3–0.6)	<0.001	0.5 (0.3–0.8)	0.003
Seldom or never	0.3 (0.2–0.6)	<0.001	0.5 (0.3–0.9)	0.021
Worry about the Mpox epidemic in China				
Yes	1.6 (1.1–2.3)	0.007	1.6 (1.1–2.3)	0.024
No	Ref		Ref	
I believe Mpox vaccines are safe for PLHIV				
Yes	11.8 (5.3–26.5)	<0.001	6.6 (2.7–16.4)	<0.001
No or not sure	Ref		Ref	
The Mpox vaccine can be effective against Mpox				
Yes	3.5 (2.2–5.4)	<0.001	1.9 (1.1–3.3)	0.021
No	Ref		Ref	

Abbreviations: ART: antiretroviral therapy; MSM: men who have sex with men; HIV: human immunodeficiency virus; PLHIV: people living with HIV; aOR: adjusted odds ratio; CI: confidence interval; Ref: reference.

## Data Availability

The data that support the findings of this study are available from the corresponding author, H.Z., upon reasonable request.

## Supplementary Materials

The following supporting information can be downloaded at: https://www.mdpi.com/article/10.3390/vaccines11030528/s1, Table S1: STROBE Statement—Checklist of items that should be included in reports of cross-sectional studies.

## Abbreviations

aOR	adjusted odds ratio
ART	antiretroviral therapy
CDC	Center for Disease Control and Prevention
CI	confidence interval
HIV	human immunodeficiency virus
MSM	men who have sex with men
PLHIV	people living with HIV
PHEIC	Public Health Emergency of International Concern
Ref	reference
WHO	World Health Organization
